# Supplementary material for: From past to present: Exploring COVID-19 in Qatar’s hemodialysis population across Omicron dominant and pre-Omicron periods
Source: PLoS One. 2023 Sep 14;18(9):e0291266. doi: 10.1371/journal.pone.0291266 (PMC10501587; doi:10.1371/journal.pone.0291266)
Supplement: Attachment — Submitted filename: Response to Reviewers comments.docx [file pone.0291266.s004.docx]

Dear Editor in Chief:

Thank you for your valuable comments. Hopefully, we have addressed all comments properly. We believe these comments led to significant improvement in our manuscript.

**Reviewers' comments:**

**A- Please ensure that your manuscript meets** **PLOS ONE's style requirements, including those for file naming.**

Thank you for your comment. We did the required modifications on the manuscript template according to PLOS ONE's style requirements.

**B- Competing Interests section.**

Thank you for your comment. We updated the competing Interests section in the revised manuscript, cover letter, online submission form, and editorial manager.

**C- Please note that in order to use the direct billing option the corresponding author must be affiliated with the chosen institute. Please either amend your manuscript to change the affiliation or corresponding author or email us at** [**plosone@plos.org**](mailto:plosone@plos.org) **with a request to remove this option.**

Thank you for your comment. Please note that the corresponding author Dr. Abdullah Hamad is affiliated with Hamad Medical Corporation, and the submission form has been updated accordingly.

**D- We note that you have stated that you will provide repository information for your data at acceptance. Should your manuscript be accepted for publication, we will hold it until you provide the relevant accession numbers or DOIs necessary to access your data. If you wish to make changes to your Data Availability statement, please describe these changes in your cover letter and we will update your Data Availability statement to reflect the information you provide**

Thank you for your comment. We uploaded the raw data (Coded Omicron VS pre Omicron Master sheet, supporting information, README.md file, IRB approval) on the PLOS1 open data with the following DOI: <https://doi.org/10.5061/dryad.9w0vt4bmn>

**E- Please amend either the abstract on the online submission form (via Edit Submission) or the abstract in the manuscript so that they are identical.**

Thank you for your comment. We amended the abstract in the revised manuscript.

**F- Your ethics statement should only appear in the Methods section of your manuscript. If your ethics statement is written in any section besides the Methods, please move it to the Methods section and delete it from any other section. Please ensure that your ethics statement is included in your manuscript, as the ethics statement entered into the online submission form will not be published alongside your manuscript.**

Thank you for your comment. The ethics statement is now included in the methods section only.

**G- We noticed you have some minor occurrence of overlapping text with the following previous publication(s),**

Thank you for your comment. We made all the necessary changes as advised. We also added two references (37 and 56), our abstracts presented in the American Society of Nephrology 2022.

**1- Please review Table 1 regarding the numbers as they do not sum up correctly and some percentages are not correct.**

Thank you for your comment. We reviewed and corrected the numbers in Table 1.

**2. There is no Table addressing the characteristics on Omicron versus non-Omicron please tabulate the data.**

Thank you for your comment. We have all characteristics in Table 1 (including demographics, co-morbidities, outcomes, etc.). If you feel we should have it separate, we will gladly do so.

**3. How did you compare CT value between both groups even though RT-PCR was not used to detect virus since early December 2021.**

Thank you for your comment. Although COVID-19 rapid antigen test was allowed to be used in the Omicron era, most patients had RT-PCR done. There were 160 out of 247 (65%) in the Omicron group, and 125 out of 174 (72%) in the non-Omicron group who had RT-PCR, and their CT values were reported. Although all 174 patients in the non-Omicron group had RT-PCR, CT values were unavailable for 49 patients, as the lab did not report CT values early during the COVID-19 pandemic. We included this information in the discussion section to clarify.

**4. You mentioned clearly in the Mortality section that Omicron related infections caused significantly higher mortality compared to non- Omicron, even though I do not see it in the Univariate and multivariate analysis table.!!**

Thank you for your comment. We clarified the sentence that death was significantly higher in the non-Omicron group at the beginning of the Mortality section. We could not do univariate or multivariate analysis to compare them as the number of deaths was relatively small (33 total, only 6 in the Omicron group).

**5. there was significant difference in all doses of vaccination between those who died and who did not, how did vaccination behave in the factor analysis.**

Thank you for your comment. We added analysis for the first vaccination dose in both univariance and multivariate analysis in the results and mortality sections and Table 3. For the second and third vaccination doses, the number of vaccinated cases in the death group was very low to perform the analysis, 9 and 5 patients, respectively.

**6. Please describe how COVID was managed in Qatar during the Omicron and on Omicron period, in ICU and general ward.**

Thank you for your comment. We added a paragraph to the discussion section, including multiple references for further reading.

24- van Teijlingen E, Sathian B, Simkhada P, Banerjee I. COVID-19 in Qatar: Ways forward in public health and treatment. Qatar Med J. 2021 Jan 4;2020(3):38. doi: 10.5339/qmj.2020.38. PMID: 33447537; PMCID: PMC7780299.

25- Barman, M.; Hussain, T.; Abuswiril, H.; Illahi, M.N.; Sharif, M.; Saman, H.T.; Hassan, M.; Gaafar, M.; Abu, J.; Ahmad, M.K.K. Embracing Healthcare Delivery Challenges during a Pandemic. Review from a nodal designated COVID-19 center in Qatar. *Avicenna* **2021**, *2021*, 8.

26- Omrani, A.S., Almaslamani, M.A., Daghfal, J. *et al.* The first consecutive 5000 patients with Coronavirus Disease 2019 from Qatar; a nation-wide cohort study. *BMC Infect Dis* **20**, 777 (2020). <https://doi.org/10.1186/s12879-020-05511-8>

27- Seedat, S., Chemaitelly, H., Ayoub, H.H., Makhoul, M., Mumtaz, G.R., Al Kanaani, Z., Al Khal, A., Al Kuwari, E., Butt, A.A., Coyle, P. and Jeremijenko, A., 2021. SARS-CoV-2 infection hospitalization, severity, criticality, and fatality rates in Qatar. *Scientific reports*, *11*(1), pp.1-10.

28- Butt AA, Dargham SR, Tang P, Chemaitelly H, Hasan MR, Coyle PV, Kaleeckal AH, Latif AN, Loka S, Shaik RM, Zaqout A, Almaslamani MA, Al Khal A, Bertollini R, Abou-Samra AB, Abu-Raddad LJ. COVID-19 disease severity in persons infected with the Omicron variant compared with the Delta variant in Qatar. J Glob Health. 2022 Jul 6;12:05032. doi: 10.7189/jogh.12.05032. PMID: 35788085; PMCID: PMC9253930.

29- Khatib MY, Ananthegowda DC, Elshafei MS, et al.: [Predictors of mortality and morbidity in critically ill COVID-19 patients: An experience from a low mortality country](https://dx.doi.org/10.1002/hsr2.542). Health Sci Rep. 2022, 5:e542. [10.1002/hsr2.542](https://dx.doi.org/10.1002/hsr2.542)

30- AlNuaimi AA, Chemaitelly H, Semaan S, et al. All-cause and COVID-19 mortality in Qatar during the COVID-19 pandemic. BMJ Global Health 2023;8:e012291. doi:10.1136/ bmjgh-2023-012291

**7.You mention use on monoclonal Ab and anti viral as possible explanation for the lower mortality in Omicron infected patients, was that standard regime of treatment of COVID during that time, how many patients received it?**

Thank you for your comment. Antiviral treatment was included in the treatment protocol of all infected dialysis patients, given they were considered a high-risk group. On the other hand, monoclonal Ab treatment was initially used in the treatment protocol but was excluded later due to lack of efficacy. We do not have the accurate number of patients who received antiviral or monoclonal antibodies treatment as our study did not investigate the treatment details.

**8. Your argument for the neutralizing Ab mounted from Omicron infection in the discussion is confusing, do you advocate that it contributed to lower mortality or not?**

Thank you for your comment. We meant that the lower titers of Omicron-specific neutralizing antibodies compared to Delta-specific neutralizing antibodies might explain the rapid spread of Omicron infection but not the effect on mortality, as the virus might evade neutralizing antibodies induced by previous infection or vaccinations. We modified the text to clarify this point.

**9. As Omicron caused much milder disease and lower mortality, on what basis do you advocate to deliver 4th booster dose.**

Thank you for your comment. We mentioned that Cinkilik et al. (39) found an improved omicron-specific humoral immunity following the fourth mRNA vaccination. Although the Omicron variant resulted in mostly mild disease, the disease might still be severe in such a vulnerable dialysis population, and a fourth booster dose might be beneficial. We modified the language to clarify the statement in the revised manuscript.

**10. The manuscript needs English language editing please.**

Extensive and professional language editing was done as advised.
