# Supplementary material for: From past to present: Exploring COVID-19 in Qatar’s hemodialysis population across Omicron dominant and pre-Omicron periods
Source: PLoS One. 2023 Sep 14;18(9):e0291266. doi: 10.1371/journal.pone.0291266 (PMC10501587; doi:10.1371/journal.pone.0291266)
Supplement: Attachment — Submitted filename: Dear Editor in Chief latest.docx [file pone.0291266.s006.docx]

Dear Editor in Chief

Thank you for your valuable comment and feedback. Please find details reply to reviewers’ comments:

1- Please review your reference list to ensure that it is complete and correct. If you have cited papers that have been retracted, please include the rationale for doing so in the manuscript text, or remove these references and replace them with relevant current references. Any changes to the reference list should be mentioned in the rebuttal letter that accompanies your revised manuscript. If you need to cite a retracted article, indicate the article’s retracted status in the References list and also include a citation and full reference for the retraction notice.

All references were checked, and none were retracted.

Style was checked and modified as needed.

2- I still have major concern about labelling the Omicron cases as you did not have definite objective method of confirming their diagnosis with the Omicron variant apart from the time variable (being diagnosed after December 2021) therefore I advise you to mention that as major limitation in your study and remove that from the title of the study as your definite results about the HD patients with COVID-19 mainly rather than Omicron versus non Omicron , and to transfer the Table of Omicron versus non Omicron to appendix due to the redundency of the inclusion criteria.

A- Your comment is very valuable and reasonable. We tried to follow lead of similar articles published in the literature (*). We agree with your assessment overall. We hope that we made the necessary changes to answer your query:

- We changed the title to reflect period of Omicron dominant period (instead of Omicron) to reflect on timing of COVID infection rather than viral gene sequencing as per your comment.

- We changes all Omicron to Omicron dominant period and We changed all non-Omicron to pre-Omicron period.

- We added it in major challenge as advised “First and most important, not all cases of Omicron dominant period are tested with genomic sequencing to prove the diagnosis. But sample testing shown that majority of COVID-19 cases in that period were of Omicron variant (24).”

- regarding table 1, as we clarified inclusion criteria (by periods not exact genomic testing). We modified title of columns to reflect that. We changed Omicron to Omicron dominant period and changed Non-Omicron to Pre-Omicron period. This table is important to reflect the changes in COVID-19 outcomes related to time (periods) and we feel it is important to the reader.

Overall, we made the changes per your advice and in similar way to describe time of infection with predominant variant rather than virus genome sequencing.

Bohnert AS, Kumbier K, Rowneki M, Gupta A, Bajema K, Hynes DM, Viglianti E, O'Hare AM, Osborne T, Boyko EJ, Young-Xu Y, Iwashyna TJ, Maciejewski M, Schildhouse R, Dimcheff D, Ioannou GN. Adverse outcomes of SARS-CoV-2 infection with delta and omicron variants in vaccinated versus unvaccinated US veterans: retrospective cohort study. BMJ. 2023 May 23;381:e074521. doi: 10.1136/bmj-2022-074521. PMID: 37220941.
