# Supplementary material for: From past to present: Exploring COVID-19 in Qatar’s hemodialysis population across Omicron dominant and pre-Omicron periods
Source: PLoS One. 2023 Sep 14;18(9):e0291266. doi: 10.1371/journal.pone.0291266 (PMC10501587; doi:10.1371/journal.pone.0291266)
Supplement: Attachment — Submitted filename: reviewer comment reply.docx [file pone.0291266.s005.docx]

Dear Editor in Chief

Thank you for your valuable comments. We did the recommended changes as advised. We hope this version will be satisfactory. Appreciate your efforts in improving our manuscript.

1. The manuscript still needs English language editing, for example “To compare characteristics of HD patients infected with COVID-19 who died to patients who stayed alive during the study and identify risk factors associated with death”
You can write it in better way : Assess characteristics of HD patients (Survivors Vs non survivors) who developed COVID-19 and identify factors associated with mortality.
Further English editing was reperformed as advised. We checked our manuscript against language editing service by AJE/Springer nature and it suggested that the paper do not need further editing (report attached with 92^nd^ percentile). Please let us know if you feel further editing needed.

2. “Per national genomic surveillance, all patients diagnosed with COVID-19 before December 1, 2021, were assigned to the non-Omicron group, while patients diagnosed on December 1, 2021, and onwards were assigned to the Omicron group”
Was the assignment to Omicron versus non omicron as per time frame or based on genomic testing of each case when diagnosed?!

Great comment. We added this short paragraph to the methods to explain with reference:

Due to the fast rise of Omicron COVID-19 infections, large scale genome sequencing was not possible in the Omicron variant (compared to previous variants), and positive cases from December 2021 onwards were considered Omicron based on representative sampling.

Butt AA, Dargham SR, Tang P, Chemaitelly H, Hasan MR, Coyle PV, Kaleeckal AH, Latif AN, Loka S, Shaik RM, Zaqout A, Almaslamani MA, Al Khal A, Bertollini R, Abou-Samra AB, Abu-Raddad LJ. COVID-19 disease severity in persons infected with the Omicron variant compared with the Delta variant in Qatar. J Glob Health. 2022 Jul 6;12:05032. doi: 10.7189/jogh.12.05032. PMID: 35788085; PMCID: PMC9253930.

3. How did you analyze the 26 patients who developed both Omicron and non Omicron variants?

Cases were analyzed based on each episode of COVID as each episode carries its own different outcomes. We added this analysis in the methodology.

4. How do you explain the same ICU length of stay between Omicron and Non Omicron infected patients even though the rate of mechanical ventilation was much higher in the Non Omicron group, why the Omicron infected patients were kept in the ICU?

Thank you for this observation. ICU admissions were due to many reasons. The percentage of patients who had mechanical ventilation out of all ICU admission were 43% in Omicron (3 out of 7) versus 63% in non-Omicron (28 out of 44). We had only a total of 7 patients in the Omicron group admitted to the ICU. Three of them were put on mechanical ventilation (table 1). The rest were put for other reasons (impending respiratory failure requiring Bipap, circulatory failure/hypotension/ sepsis). Will be happy to add more details in the discussion section if you deem necessary. Once there were in ICU, length of stay was similar as both groups had severe illness requiring admission.

5. What do you mean by univariate analysis in Table 3 and what is the difference between it and the multivariate analysis next to it and the difference with Table 2, and describe this in the methodology.

Thank you for this valuable comment. We added a paragraph in the methods to explain this in details.

6. You mentioned the incidence of Omicron versus Non omicron variant infection in the discussion. How did you calculate it, and where is that information in the results section?

Thank you for this observation. We agree with you. We added method of calculation in the methods section and outcome in the result section.

7. I don’t understand why do you mention stressfully the relation between CT value and outcome and correlate that with the Omicron values, even though the CT value was not statistically significant factor associated with mortality in the bivariate and multivariate analysis and the references you refer to do not give strong prediction of what you are proposing.

We agree with your comment. We rephrased the sentence to represent our significant outcomes only.

8. I still find your advocacy for the fourth dose of vaccine in HD patients specifically and in general is not strongly explained in the discussion part, as the available vaccines produce low level of Omicron-specific neutralizing antibodies, compared to other variants, and the Omicron variant is associated with very much mild disease, and facing the current worldwide discussion of possible side effects from COVID-19 vaccines that were fast tracked with now properly done studies, the fourth or subsequent vaccination for Omicron variant is not strongly justified.

Agree and removed this part from discussion.

9. Still you don’t explain the low mortality reasons in Omicron cases clearly in the discussion, once you claim it is the use of neutralizing Ab and then the antiviral, then the reception of booster vaccine dose (even though you claim that Omicron-specific neutralizing antibodies are not well mounted by vaccination), please improve the fidelity and strength of your discussion in that part.

Thank you for this important remark. We modified the discussion to highlight that transmission is higher in Omicron and potential causes (including evasion of vaccine), but we stressed that despite this evasion, there is still relative protection provided by the vaccine (especially with adding 3^rd^ dose) compared to unvaccinated patients (and that include reducing transmission and morbidity). We added references too.
